# Simultaneous venous–arterial Doppler during preload augmentation: illustrating the Doppler Starling curve

**DOI:** 10.1186/s13089-023-00330-9

**Published:** 2023-07-28

**Authors:** Jon-Émile S. Kenny, Stanley O. Gibbs, Joseph K. Eibl, Andrew M. Eibl, Zhen Yang, Delaney Johnston, Chelsea E. Munding, Mai Elfarnawany, Vivian C. Lau, Benjamin O. Kemp, Bhanu Nalla, Rony Atoui

**Affiliations:** 1 Health Sciences North Research Institute, Sudbury, ON Canada; 2 Flosonics Medical, 325 W. Front Street, Toronto, ON Canada; 3 NOSM University, Sudbury, ON Canada; 4 OSF Saint Francis Medical Center, Peoria, IL USA

**Keywords:** Fluid responsiveness, Fluid tolerance, Carotid Doppler, Venous Doppler, Functional hemodynamic monitoring, Passive leg raise

## Abstract

Providing intravenous (IV) fluids to a patient with signs or symptoms of hypoperfusion is common. However, evaluating the IV fluid ‘dose–response’ curve of the heart is elusive. Two patients were studied in the emergency department with a wireless, wearable Doppler ultrasound system. Change in the common carotid arterial and internal jugular Doppler spectrograms were simultaneously obtained as surrogates of left ventricular stroke volume (SV) and central venous pressure (CVP), respectively. Both patients initially had low CVP jugular venous Doppler spectrograms. With preload augmentation, only one patient had arterial Doppler measures indicative of significant SV augmentation (i.e., ‘fluid responsive’). The other patient manifested diminishing arterial response, suggesting depressed SV (i.e., ‘fluid unresponsive’) with evidence of ventricular asynchrony. In this *short communication*, we describe how a wireless, wearable Doppler ultrasound simultaneously tracks surrogates of cardiac preload and output within a ‘Doppler Starling curve’ framework; implications for IV fluid dosing are discussed.

## Main text

### Introduction

The prescription of intravenous (IV) fluid is a clinical decision most often triggered by signs and symptoms of organ hypoperfusion and guided by traditional vital signs [1, 2]. However, the intended physiological effect of IV fluid (i.e., to augment stroke volume (SV) [3]) is rarely measured, especially in the emergency department [4]. Importantly, widely employed clinical markers such as urine output and traditional vital signs do not reliably indicate blood flow response [5]; therefore, without directly quantifying SV, the intended effect of cardiac preload is inscrutable.

In early sepsis and septic shock, a clinically significant proportion of patients receiving IV fluids do not exhibit SV augmentation and, as resuscitation progresses, more than 90% of patients cease having the anticipated SV-enhancing effect of a crystalloid infusion [6–8]. Importantly, withholding IV fluids in patients who fail to have the desired physiological effect is not harmful [6]. In fact, in a separate randomized and controlled trial, guiding IV fluids by changing SV (SV_∆_) led to significantly less IV fluid administered and improved patient-centered outcomes [9].

We have described a novel, wearable Doppler ultrasound that measures and displays common carotid artery and internal jugular Doppler spectrograms [10–16]. These real-time data afford synchronously acquired surrogates of changing SV and right atrial pressure, respectively [12, 13, 17–19]. Indeed, to our knowledge we were the first to describe simultaneous venous and arterial Doppler assessments during a passive leg raise (PLR) maneuver in a critically ill patient [12]. Further, a recent physiological framework based on this data elaborated the link between real-time venous and arterial Doppler [20]. In this *short communication*, we describe two patients receiving preload augmentation in an emergency department (ED) while monitored by the wearable Doppler; clinical and physiological implications are considered within the framework of a ‘Doppler Starling curve’.

### Patients and consent

Written and informed consent was obtained for both patients for publication of this report and accompanying images; the study was approved by the Peoria Institutional Review Board. The study was performed in accordance with the ethical standards as laid down in the 1964 Declaration of Helsinki and its later amendments. Both patients were recruited from a community emergency department and were deemed in need of IV fluid resuscitation by the treating clinician who was blinded to the results of the wearable ultrasound. The choice for preload augmentation was at the discretion of the treating clinician.

### Wearable Doppler ultrasound

**Fig. 1:**
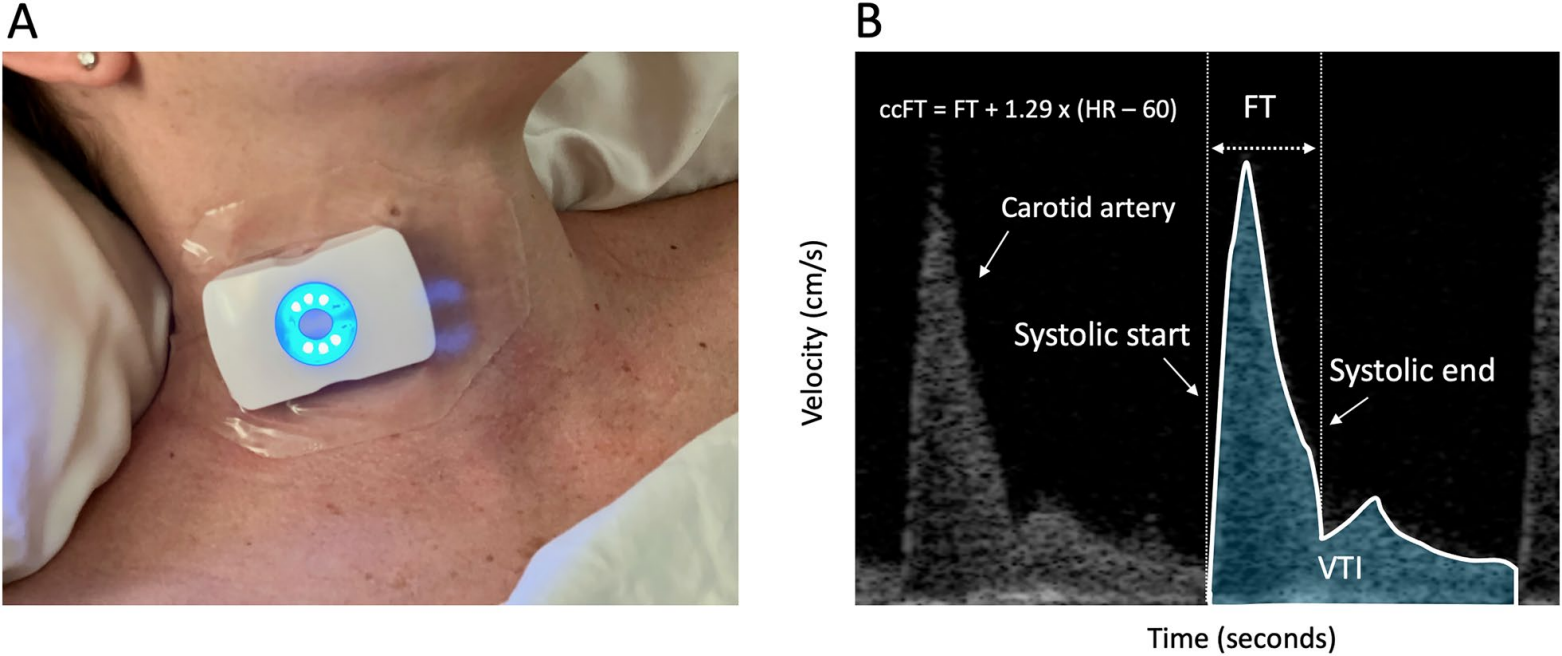
The wireless, wearable Doppler biosensor. **A** The device on a healthy volunteer. **B** The carotid artery Doppler spectrogram with the area under the velocity time curve, or velocity time integral (VTI), and flow time (FT) demarcated by the onset and end of systole. The FT is corrected for heart rate by the equation of Wodey to give the carotid corrected flow time (ccFT). The VTI for a single cardiac cycle represents the distance traveled by the blood, in centimeters, per beat. The ccFT is the duration of mechanical systole in milliseconds, corrected for heart rate by the equation of Wodey [22]

The ultrasound patch (Flosonics Medical, Sudbury, ON. Canada) is a wearable, wireless, FDA-cleared, continuous-wave 4 MHz ultrasound. Adhesive straps fix the transducer angle relative to the direction of carotid blood flow [21]. The wearable ultrasound displays real-time carotid corrected flow time (ccFT) as well as the common carotid velocity time integral (VTIc) (Fig. 1).

### Doppler analysis

The Doppler spectrograms were analyzed for the absolute and % change in ccFT (ccFT_∆_) and % change in VTI (VTIc_∆_). The number of cardiac cycles averaged before and during preload augmentation was dictated by the coefficient of variation of each particular measure to ensure change could be detected with statistical confidence [23]. The assessment windows showing the largest change between baseline and preload augmentation were considered for analysis. The primary determinant of whether or not a patient was deemed ‘preload responsive’ was the threshold reported by Barjaktarevic and colleagues, that is, + 7 ms ccFT_∆_ [24]. We also considered, as secondary determinants, the thresholds that we have identified during simulated severe hypovolemia and blood transfusion in healthy volunteers, that is +4% ccFT_∆_ and +18% VTIc_∆_ [14, 15].

**Fig. 2:**
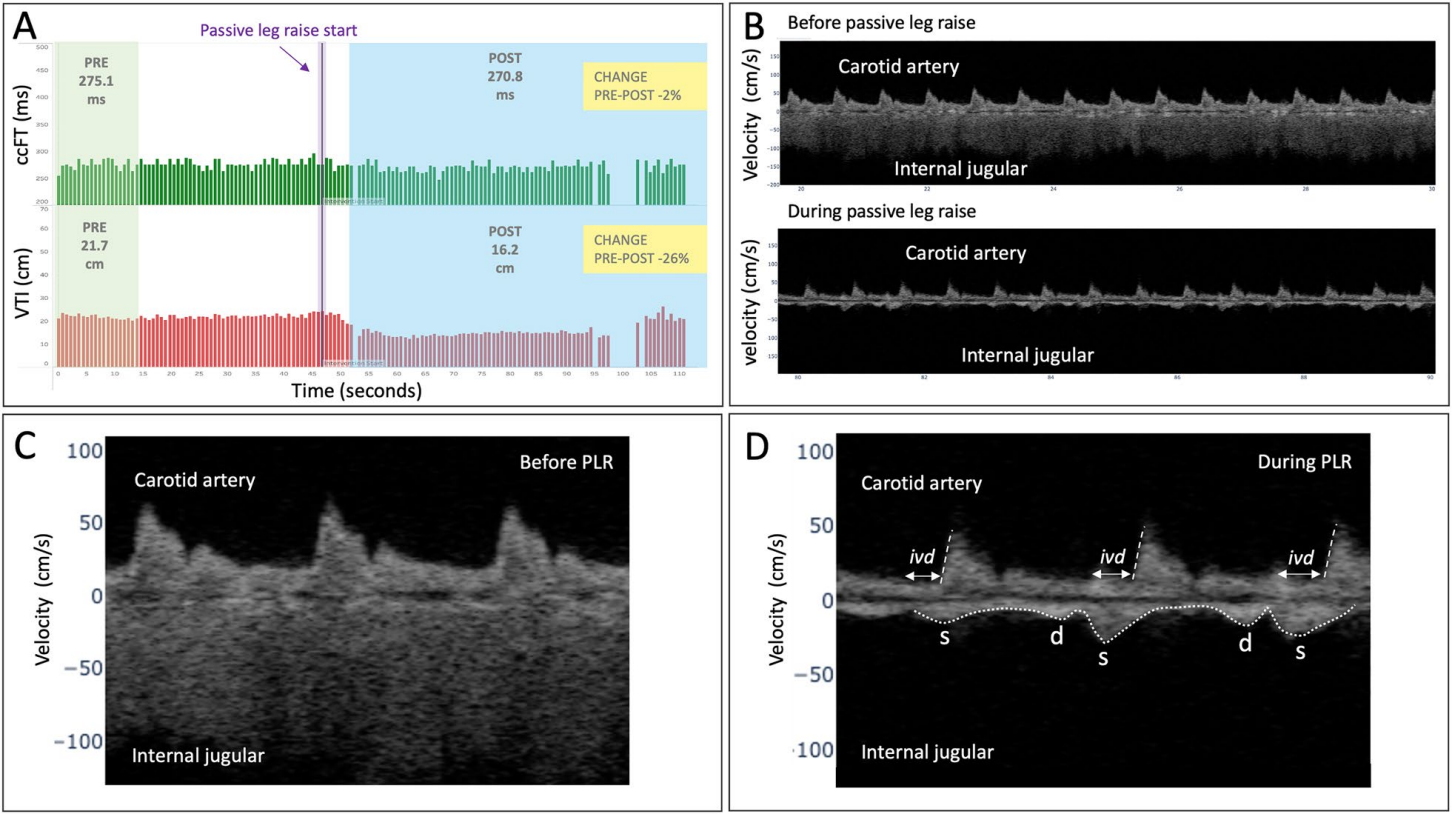
The results of the passive leg raise maneuver in patient 1. **A** The user display of the wearable Doppler ultrasound. The green bars represent the ccFT per cardiac cycle, the red bars the VTIc per cardiac cycle. The green-shaded window labeled ‘pre’ averages all cardiac cycles within the window. The vertical, purple line is where the leg raise begins. The blue-shaded ‘post’ window during the PLR averages all cardiac cycles within the window and is compared to the ‘pre’ value to calculate change. The window size is based on the coefficient of variation and can be moved by the clinician. **B** Two strips of the carotid and jugular spectrograms above and below the x-axis, respectively, before and during the leg raise. **C** 3 cardiac cycles before the leg raise with higher resolution. The jugular velocity is high, and the amplitude (brightness) is low, suggesting a collapsed vein. **D** 3 cardiac cycles during the leg raise. The venous Doppler velocity falls and the amplitude (brightness) increases—suggesting a distended vein. The venous velocity becomes pulsatile with venous systole (‘s’ wave) preceding arterial systole (carotid upstroke) illustrating interventricular delay (‘ivd’) consistent with the patient’s known incomplete left bundle branch block. Compare the temporal relationship of the venous ‘s’ and ‘d’ waves with patient 2

**Fig. 3:**
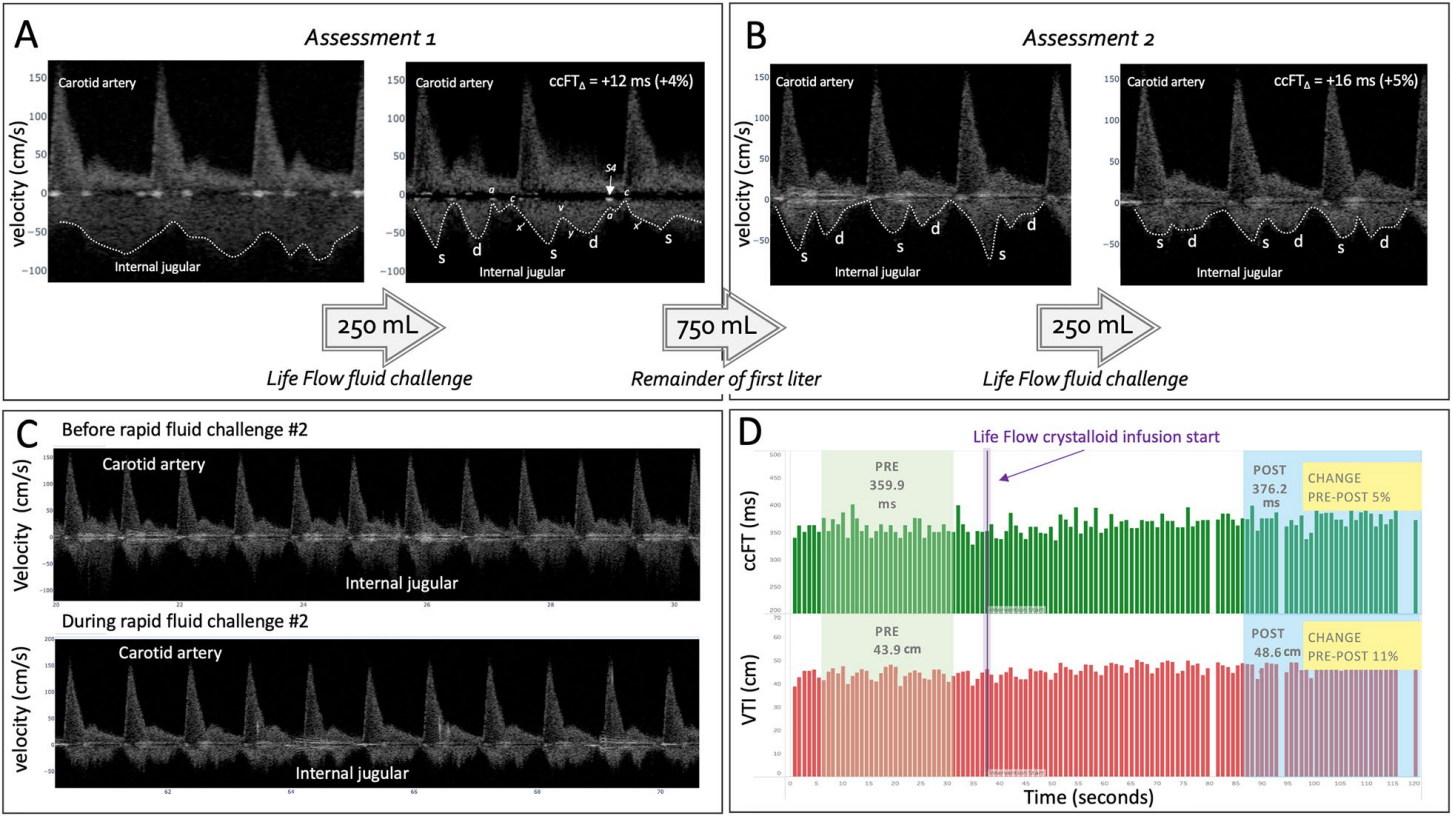
The results of the rapid fluid challenges (assessments) in patient 2. **A** Three cardiac cycles from before and during the first rapid fluid challenge; the venous morphology evolves from higher velocity, continuous to pulsatile, ‘s’ > ‘d’ wave which follows the right atrial pressure trace. Briefly, as the jugular vein changes from a collapsed, ellipsoid structure in cross-section, the Doppler spectrogram changes from a higher velocity, minimally undulating morphology into a pulsatile pattern that adopts the right atrial pressure waveform. Atrial kick, ‘a’ wave, creates a velocity minimum at end-diastole and may be accompanied by a visible S4 on the spectrogram. This is followed by the x’-descent, generating the venous Doppler systolic ‘s’ wave. The pooling venous blood in the right atrium as systole progresses creates the v wave in the pressure waveform; this corresponds to a velocity minimum that cleaves the venous Doppler into the ‘s’ and the diastolic ‘d’ wave at the onset of the y-descent (tricuspid valve opening). With rising right atrial pressure and/or tricuspid regurgitation, the x’-descent magnitude shrinks relative to the y-descent; thus, the Doppler ‘s’ wave falls relative to the ‘d’ wave which can (not pictured) lead to only diastolic filling. The ccFT increases significantly (+ 12 ms). **B** Three cardiac cycles before and during the second fluid challenge, following 1 L crystalloid. The jugular velocity is biphasic; the ‘s’ wave greater than the ‘d’ wave. With the rapid fluid challenge, the ‘s’ wave velocities fall, the ccFT again increases (+ 16 ms). **C** Two longer recordings before and during the second fluid challenge. **D** The entire second rapid fluid challenge. The green bars represent the ccFT per cardiac cycle, the red bars the VTIc per cardiac cycle. The green-shaded window labeled ‘pre’ is the average of all cardiac cycles within the window. The vertical, purple line is where the rapid fluid challenge begins. The blue-shaded window during the fluid challenge averages all cardiac cycles needed to calculate change with statistical significance; the window size is based on the coefficient of variation

The venous Doppler spectrograms were analyzed qualitatively based on the framework put forth by us [25] and Iida and colleagues [26]. These venous spectrogram changes have been previously described in the jugular vein [27–29], superior vena cava [30], inferior vena cava [31], hepatic veins [32] and even the femoral vein [33]. Use of the hepatic, portal and intra-renal venous Doppler have been incorporated into the recently-described venous excess ‘VExUS’ score [34] (see Figs. 2, 3). Venous Doppler data were evaluated every 30 s before, during and after the preload challenge to assess for morphological changes consistent with rising right atrial (or central venous) pressure.

### Preload augmentation

Both patients had baseline measures recorded in the semi-Fowler position at 45 degrees. Prior to preload augmentation, at least 30 s of continuous Doppler spectrograms were acquired with the patient instructed to remain motionless and breathe quietly. Patient 1 received a PLR by a clinical assistant lifting the legs to increase cardiac preload; the Doppler spectrograms were monitored continuously while the patient was supine with legs raised for an additional 60 s. For patient 2, the first 250 mL of each liter ordered comprised a ‘rapid fluid challenge’ (RFC) assessment. The 250 mL RFC was administered at a rate of 100 mL/min while the patient remained in semi-Fowler [35, 36]. This rate of fluid infusion was achieved using the LifeFlow (Durham, N.C., U.S.A.) device which can provide IV bolus rates upwards of 250 mL/min through a peripheral IV. During the RFC assessment, Doppler spectrograms were monitored continuously for an additional 180 s, thus recording the entire 250 mL of rapid saline infusion plus an additional 30 s of monitoring after the rapid infusion ended. The remaining 750 mL of each liter ordered were infused at the discretion of the treating clinician.

### Patient 1

A 72-year-old man presented to the emergency department with altered mental status, back pain, hypotension, leukocytosis, acute kidney injury and a working diagnosis of sepsis due to lumbar osteomyelitis versus ascending urinary tract infection. He had a known history of tricuspid and mitral band valvuloplasties and diminished left ventricular ejection fraction (LVEF) of 40% consequent to chronic hypertension and ethanol use. His outpatient medications were lisinopril, furosemide and carvedilol. The patient’s intake vital signs were a heart rate of 83 beats per minute, blood pressure of 69/52 mmHg, afebrile with normal respiratory rate and oxygen saturation. His ECG showed a possible ectopic atrial rhythm with incomplete left bundle block pattern. In the emergency department, a PLR was performed prior to IV fluids and the results presented in Table 1 and Fig. 2.

**Table 1:** Carotid artery Doppler measures

Carotid Doppler measure	ccFT coefficient of variation	Cardiac cycle sample window	Absolute ccFT_∆_	% ccFT_∆_	% VTIc_∆_
Patient 1	3.3%	19 cardiac cycles	− 4.31 ms	− 2.0%	− 26.0%
Patient 2	4.1%	28 cardiac cycles	+ 16.3 ms	+ 5.0%	+ 11.0%

The coefficient of variation is the standard deviation divided by the mean of the baseline section, this was used to determine the number of cardiac cycles in the baseline and intervention periods needed to detect change with statistical confidence. The ccFT_∆_ and VTIc_∆_ for patient 2 but not patient 1 are consistent with a clinically significant increase in stroke volume [15, 24]

Patient 1 was treated with broad spectrum antibiotics and required norepinephrine infusion to maintain a mean arterial pressure above 60 mmHg. He was transferred to the intensive care unit where trans-thoracic echocardiography showed abnormal septal motion with systolic and diastolic flattening consistent with right ventricular (RV) pressure and volume overload, dilated RV size and reduced RV systolic function with RV hypertrophy. The patient had no significant valvular abnormalities, indeterminate left ventricular (LV) diastolic function, and a left ventricular ejection fraction (LVEF) of 50% while on norepinephrine.

### Patient 2

A 54-year-old woman presented with shortness of breath and acute hypercapnic and hypoxemic respiratory failure secondary to sepsis from an infected sacral ulcer versus urinary source. She had history of traumatic quadriplegia with chronic sacral wounds complicated by osteomyelitis, indwelling suprapubic catheter, active cigarette smoker, COPD on home oxygen, diastolic dysfunction with congestive heart failure, obstructive sleep apnea and type II diabetes mellitus. She was febrile on presentation with a heart rate of 75 beats per minute, blood pressure 87/51 mmHg, respiratory rate 25 breaths per minute and 93% on 6 L nasal cannula. In the ED she received broad spectrum antibiotics. The patient had a Doppler assessment performed for the first 250 mL of each of the two liters received in the ED. Given her hypotension, the first and second liters were given in rapid succession (Table 1, Fig. 3).

This patient was briefly initiated on norepinephrine after the second liter and admitted to the intensive care unit. Trans-thoracic echocardiography in the ICU revealed a dilated right ventricle (RV) and inferior vena cava (IVC) with normal RV systolic function and no valvular abnormalities; the LVEF was 60–65% with normal diastolic function.

## Discussion

**Fig. 4:**
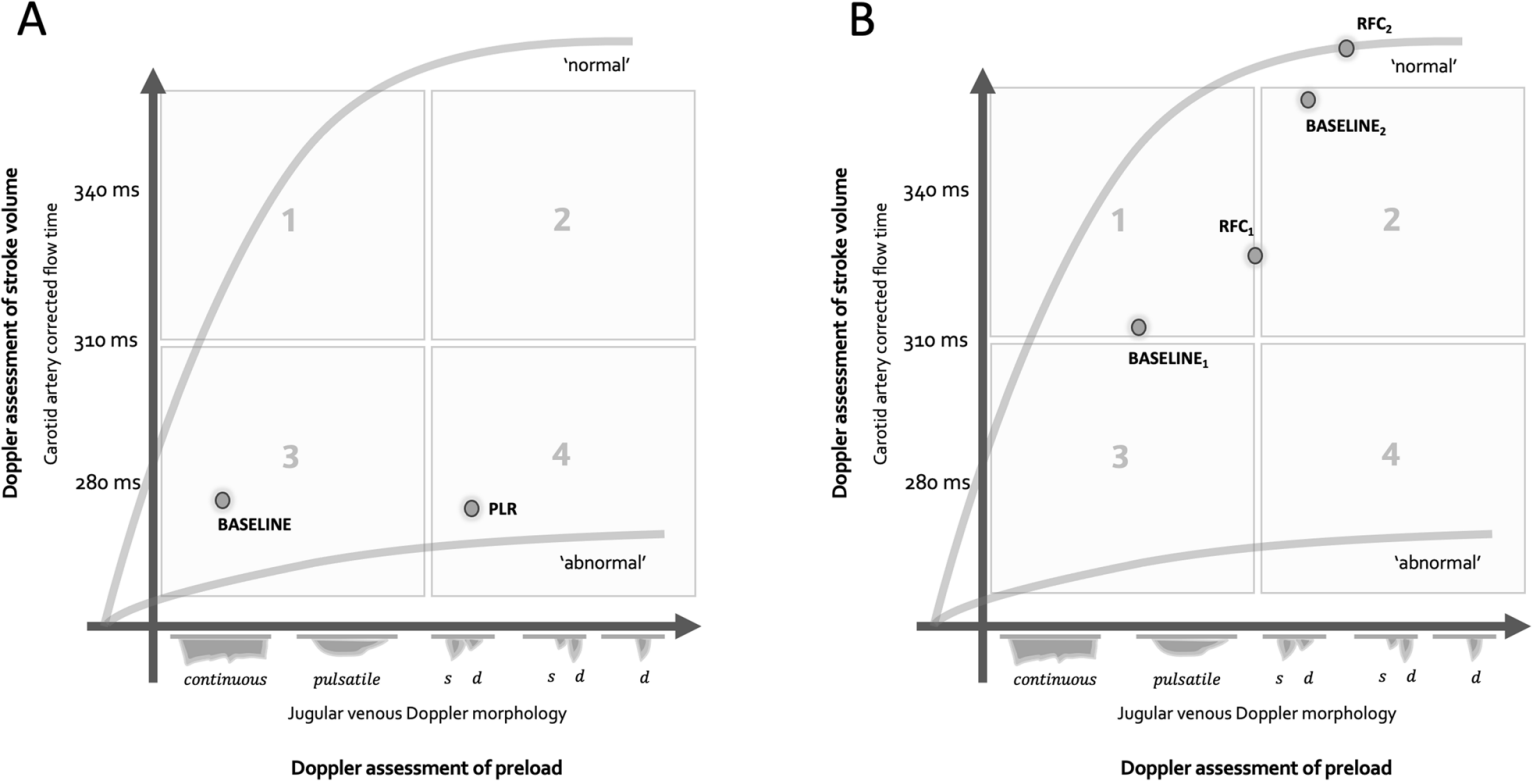
The ‘Doppler Starling curve’. The ‘normal’ and ‘abnormal’ curves represent extremes of cardiac function. **A** patient 1 begins in quadrant 3 with jugular Doppler spectrogram consistent with low central venous pressure; there is also low ccFT (baseline). With passive leg raise (PLR), the jugular spectrogram changes with rising central venous pressure, but ccFT falls. **B** patient 2 begins in quadrant 1 (baseline_1_); the first rapid fluid challenge (RFC_1_) changes both jugular venous and carotid arterial spectrograms in a manner consistent with rising central venous pressure and stroke volume, respectively. Baseline_2_ and RFC_2_ show the effects of the second fluid challenge

The baseline, low power, non-pulsatile, high velocity venous Doppler spectrograms observed in both patients strongly suggested jugular vein collapse, indicating low CVP (i.e., < 5 mmHg) [25, 37, 38]. Nevertheless, the response of the arterial Doppler spectrogram for patient 1 during PLR revealed a state of ‘fluid unresponsiveness’, or perhaps even a detrimental response to preload (Fig. 4). Accordingly, patient 1 began in ‘quadrant 3’ [20] where his low, baseline CVP is potentially misleading with regard to IV fluid provision. That is to say, this patient disclosed preload intolerance only when a dynamic maneuver (e.g., PLR) was executed; thus, the ‘quadrant 3’ physiology illustrated is a state of *dynamic fluid intolerance* [20].

Recognizing a state of dynamic fluid intolerance is important clinically because many of these patients might receive physiologically unhelpful preload based upon markers of right heart filling. For example, a CVP of less than 5 mmHg was observed in 25% of fluid unresponsive patients [39], while in a meta-analysis, a CVP of less than 8 mmHg was observed in upwards of 40% of fluid unresponsive patients [40]. Similar physiology plays out with assessments of inferior vena cava size and collapsibility [41]. As might be anticipated, holding IV fluids in unresponsive patients (e.g., patient 1) does not have adverse effects in sepsis and septic shock [6, 42]. Furthermore, when IV fluid is guided by measures of ‘fluid responsiveness’, one multicenter, randomized and controlled trial in sepsis and septic shock showed that patients received less IV fluid and had improved outcomes [9]. Given that hundreds of millions of liters of IV fluid are administered every year in the United States alone [43], holding physiologically ineffective IV fluids might lead to significant cost-savings at the population level [16, 42, 44].

That the arterial Doppler response of patient 1 implied falling SV during the PLR deserves some elaboration, albeit speculative. Given the results of his trans-thoracic echocardiogram (TTE) performed within hours after his assessment by the wearable Doppler ultrasound, both diastolic and systolic ventricular interdependence likely contributed to impaired LV output. That is, with RV volume overload noted on the TTE, RV diastolic filling during the PLR diminished LV preload via cross-ventricular diastolic stiffening [45]. Further, given RV pressure overload, systolic ventricular interaction may have additionally impaired the LV. This is especially noteworthy given the dynamic, mechanical asynchrony observed by the wearable Doppler induced by the PLR. With increased preload, the onset of RV systole (i.e., venous ‘s’ wave) occurred significantly before the carotid upstroke. As the carotid waveform temporally reflects LV systole, the interval between the venous ‘s’ wave and carotid upstroke illustrates a dynamic, mechanical interventricular conduction delay (i.e., ‘ivd’, Fig. 2D)—consistent with the patient’s known incomplete left bundle branch block. When this occurs, RV systolic ejection stiffens the interventricular septum prior to LV ejection and generates unfavorable mechanics (including worsened functional mitral regurgitation) for the delayed LV contraction [46, 47]. Indeed, this pathophysiology is the rationale for ventricular resynchronization with biventricular pacing [48]. To our knowledge, this report is the first demonstrating dynamic, mechanical ventricular asynchrony using simultaneous venous and arterial Doppler.

The primary limitation of this *short communication* is the small, clinical sample size, though this report is not meant to change local clinical practice or protocols. Our objective is to highlight bedside physiology with a novel and potentially helpful clinical biosensor. In both patients neither CVP nor SV was measured, so the data from the wearable Doppler are inferences. Nevertheless, data supporting ccFT_∆_ as a surrogate for SV_∆_ have good clinical evidence [49] and we have shown that VTIc_∆_ correlates strongly with SV_∆_ in the largest-known Doppler data set making this comparison [14]. Furthermore, in a proof-of-principle description of two patients monitored with trans-esophageal echocardiography, the optimal VTIc_∆_ for detecting significant SV_∆_ mirrored that in our healthy volunteer studies [10]. Nevertheless, carotid artery distention with IV fluids could dissociate flow from VTI and carotid flow itself is a surrogate for left ventricular output only when the ratio of total body impedance-to-downstream carotid impedance remains relatively constant [15, 50].

## Conclusions

In this *short communication*, we described the clinical application of a novel, wearable, Doppler biosensor in two patients deemed to need IV fluids by clinical examination. Simultaneous venous and arterial Doppler spectrograms are plotted using a novel framework best characterized as a “Doppler Starling curve”. Both patients initially demonstrated venous Doppler waveforms consistent with low CVP, which increased during preload augmentation. However, only patient 2 exhibited simultaneously acquired arterial Doppler changes indicative of rising stroke volume. By contrast, patient 1 displayed dynamic fluid intolerance; evidence from large, randomized controlled trials suggests that this phenotype may be managed by withholding additional IV fluids, ostensibly preventing downstream complication and cost.

## Data Availability

The datasets used and/or analyzed during the current study are available from the corresponding author on reasonable request.

## Abbreviations

IV: Intravenous
SV: Stroke volume
SV_∆_: Change in stroke volume
CVP: Central venous pressure
PLR: Passive leg raise
ED: Emergency department
FDA: Food and Drug Administration
ccFT: Corrected carotid flow time
ccFT_∆_: Change in corrected carotid flow time
VTIc: Velocity time integral of the carotid artery
VTIc_∆_: Change in the velocity time integral of the carotid artery
VTI: Velocity time integral
FT: Flow time
RFC: Rapid fluid challenge
mL/min: Milliliters per minute
mL: Milliliters
ms: Milliseconds
LVEF: Left ventricular ejection fraction
Ivd: Interventricular delay
RV: Right ventricular
LV: Left ventricular
IVC: Inferior vena cava
VExUS: Venous excess ultrasound score
TTE: Transthoracic echocardiography
